# Familial Aplasia Cutis Congenita With Exposure of the Meningeal Membrane: A Case Report

**DOI:** 10.7759/cureus.115515

**Published:** 2026-08-31

**Authors:** Leandro R Kuniyoshi, Jessica B Pozza, Rodrigo C Barreiros, Karem C Silva, Marta W Vieira

**Affiliations:** 1 Pediatrics, Pontifícia Universidade Católica de São Paulo (PUC-SP), Faculty of Medical and Health Sciences, Sorocaba, BRA; 2 Neonatology, Pontifícia Universidade Católica de São Paulo (PUC-SP), Faculty of Medical and Health Sciences, Sorocaba, BRA; 3 Dermatology, Pontifícia Universidade Católica de São Paulo (PUC-SP), Faculty of Medical and Health Sciences, Sorocaba, BRA

**Keywords:** aplasia cutis congenita acc, aplasia cutis congenita management, congenital diseases, congenital skin aplasia, conservative and surgical treatment

## Abstract

Aplasia cutis congenita (ACC) is a rare congenital malformation characterized by the localized absence of skin and occasionally subcutaneous and bone tissues. Its etiology is multifactorial, and management ranges from conservative wound care to surgical intervention. We report the case of a male newborn presenting with a 4-5 cm ulcerated lesion at the vertex of the scalp, with complete absence of bone tissue and dural exposure. Physical examination revealed syndromic features (facial asymmetry, hypertelorism, low-set ears, and cutaneous syndactyly) and an atrial septal defect. A strong autosomal dominant family history of ACC was also noted. The case described best fits Frieden’s type 9 classification. The presentation strongly suggests Scalp-Ear-Nipple syndrome (SENS), a genetic disorder linked to Potassium Channel Tetramerization Domain 1 (KCTD1) mutations. Chromosomal anomalies, such as Patau syndrome, were clinically and genetically excluded. While the lesion's extent and meningeal exposure could warrant surgical grafting according to some references, a review of the literature supports prioritizing conservative management to avoid severe anesthetic, hemorrhagic, and infectious risks in the neonatal period. This case highlights the importance of detailed clinical and familial evaluation to diagnose syndromic ACC when genetic sequencing is inaccessible. It also reaffirms that conservative management is a safe and effective alternative to surgery, even for extensive lesions with dural exposure.

## Introduction

First described in 1767 by Cordon, aplasia cutis congenita (ACC) is a rare malformation characterized by the partial or complete absence of skin, which may also affect subcutaneous tissues and bones [1]. It’s a rare condition, with a prevalence of 4,81 in 100,000 live births, and has a mortality rate ranging from 20% to 55%, depending on the extent of the lesion, other congenital anomalies, and associated complications [2,3]. Clinically, it manifests as superficial or deep lesions, ulcerated or well-demarcated, containing granulation or scar tissue. The extent is variable, ranging from small areas to lesions that affect about 90% of the body surface, with or without the involvement of extremities [2,4]. The most affected region is the scalp (85% of cases); among these, only 35% involve underlying structures, such as the subcutaneous tissue and the skull [1,5].

The etiology and pathophysiology of ACC are multifactorial and cannot be reduced to a single cause. The condition may be related to risk factors during pregnancy, such as exposure to drugs (cocaine, heroin, and alcohol), use of teratogenic medications, trauma, uterine vascular alterations, neural tube closure defects, and congenital infections, notably the herpes and varicella viruses [3,5,6]. Furthermore, ACC may be associated with several genetic syndromes, such as Adams-Oliver, Setleis, Anderson-Hollister-Szalay, Wolf-Hirschhorn syndrome, Patau, and Scalp-Ear-Nipple syndrome (SENS) [3,7,8]. Specifically, regarding SENS, a recent study by Raymundo et al. found that the deletion of Potassium Channel Tetramerization Domain 1 (KCTD1) (Ch18q11.2) and KCTD15 (Ch19q13.32), proteins associated with the syndrome, can result in ACC [9].

Although most cases do not have a defined primary cause, there are reports of autosomal dominant and, more rarely, recessive inheritance [3,5]. A European epidemiological study, which analyzed more than 10 million births, showed that hereditary factors account for only 2% of the incidence [2]. Given the multiplicity of clinical presentations, Frieden proposed, in 1986, a classification that encompasses the diverse phenotypes and etiologies of ACC, which remains in use to this day [1,6].

Diagnosis can be made during the intrauterine period, through obstetric ultrasounds, or immediately at birth, upon visual confirmation of the lesions. Although the recognition of the malformation is straightforward, it is essential to perform complementary exams to screen for possible associated malformations, such as meningocele, congenital heart defects, duodenal atresia, glaucoma, cryptorchidism, limb anomalies, and renal malformations, in addition to investigating correlated syndromes. The therapeutic management is divided into two main approaches: conservative (expectant) and surgical. Both present risks and limitations: conservative management can expose the patient to a higher chance of infections, necrosis, and hemorrhages; surgical intervention involves the inherent risks of general anesthesia, infection, hemorrhage, and eventual loss or necrosis of the graft. Therefore, the choice of treatment must be individualized, considering crucial clinical factors such as the size, depth, and location of the lesion [3,5].

Therefore, the purpose of this article is to report a case of ACC located on the vertex of the scalp, with involvement of subcutaneous tissues and the cranial vault, highlighting the possibility of conservative management even for extensive lesions with dural exposure. Furthermore, the relevance of the family history is highlighted: the newborn is the child of a father previously diagnosed with ACC and has a brother with the same malformation, which suggests an important familial component in this case. 

## Case presentation

The patient is a male newborn of 37 weeks and five days, born to non-consanguineous parents, who was admitted with a “scalp malformation" on the first day of life. The newborn was delivered by cesarean section due to breech presentation, showing good vitality, an Apgar score of 9 in the first minute and 10 at minute five, a birth weight of 3,100 g, 48 cm in length, a head circumference of 34 cm, and a chest circumference of 32 cm.

The newborn is the third child of a 32-year-old healthy mother, G3P2, and the second child of a 35-year-old father. The mother, who received adequate prenatal care, denied pathologies during pregnancy and usage of teratogenic drugs or illicit substances. Maternal serologies for toxoplasmosis, syphilis, cytomegalovirus, and rubella were all negative. No abnormalities were found in prenatal ultrasound tests, and the mother was not exposed to radiation during pregnancy. The father was reported to have had ACC at birth, as did his first child, now 10 years old, and one of his cousins, who died a few hours after she was born. The cause of death is unknown to the family. Both father and son received conservative treatment and presented no other malformations. There are no reports of genetic syndromes in their families, and an exome sequencing test couldn’t be performed due to socioeconomic issues. After birth, an ulcerated lesion at the vertex of the scalp was noticed, measuring 4-5 cm in extension, with exposure of dura mater. No drainage of cerebrospinal fluid, bleeding, or secretions was found (Figure 1). The patient was referred to a specialized service for management of the lesion and investigation of associated malformations.

**Figure 1:**
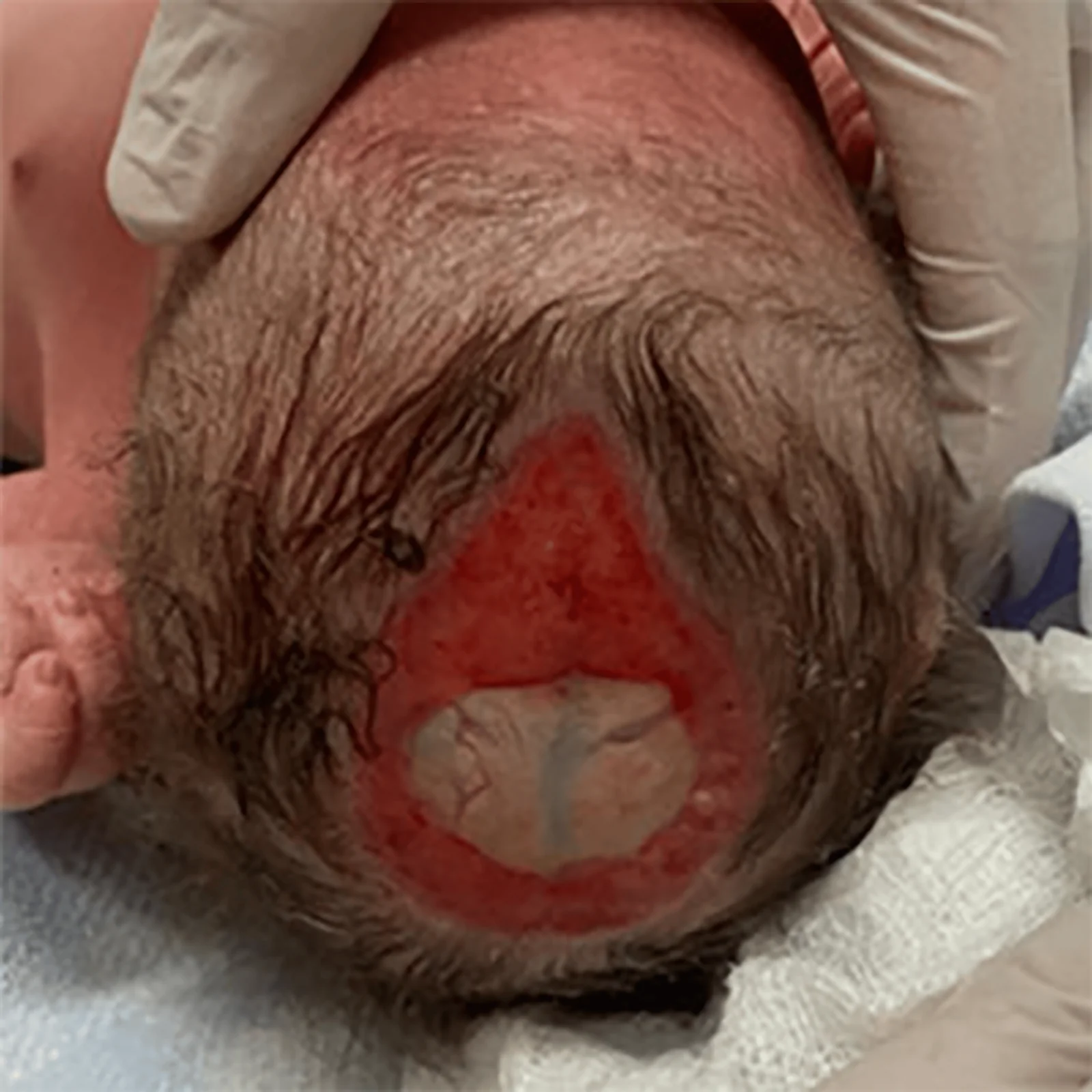
Ulcerated wound that was noticed at birth It measures 4-5 cm in extent, with complete absence of the skin, subcutaneous tissue, and bone at the vertex of the scalp, resulting in the exposure of the meningeal membrane

On physical examination, in addition to the ulcer, the newborn had small, low-set, slightly posteriorly rotated ears; ocular and mammary hypertelorism; facial asymmetry; and partial syndactyly between the second and third toes of the right foot, which X-ray imaging later revealed to be cutaneous syndactyly. No abnormalities were found during cardiac and respiratory auscultation. The suction, palmar and plantar grasp, plantar reflex, Moro, tonic-neck, and propulsion reflexes were all present, without exacerbations. There was no evidence of motor deficits or other neurological alterations. A karyotype test was performed, which excluded chromosomal abnormalities.

The computed tomography (CT) scan of the skull, taken 24 hours after birth, demonstrated an absence of bone formation at the vertex of the scalp, with a widening of the sagittal fissure and forming a communication of the fontanelles (Figure 2). There was no radiological imaging evidence of encephalic involvement. Ultrasound of the abdomen and urinary tract was requested in order to rule out associated congenital malformations, but they showed no abnormalities. A transthoracic echocardiogram (TTE) was performed five days after birth, revealing a 4 mm atrial septal defect, without clinical repercussions. Considering the family history of the pathology and its conservative management, healing by secondary intention was the chosen approach for the newborn (Figure 3).

**Figure 2:**
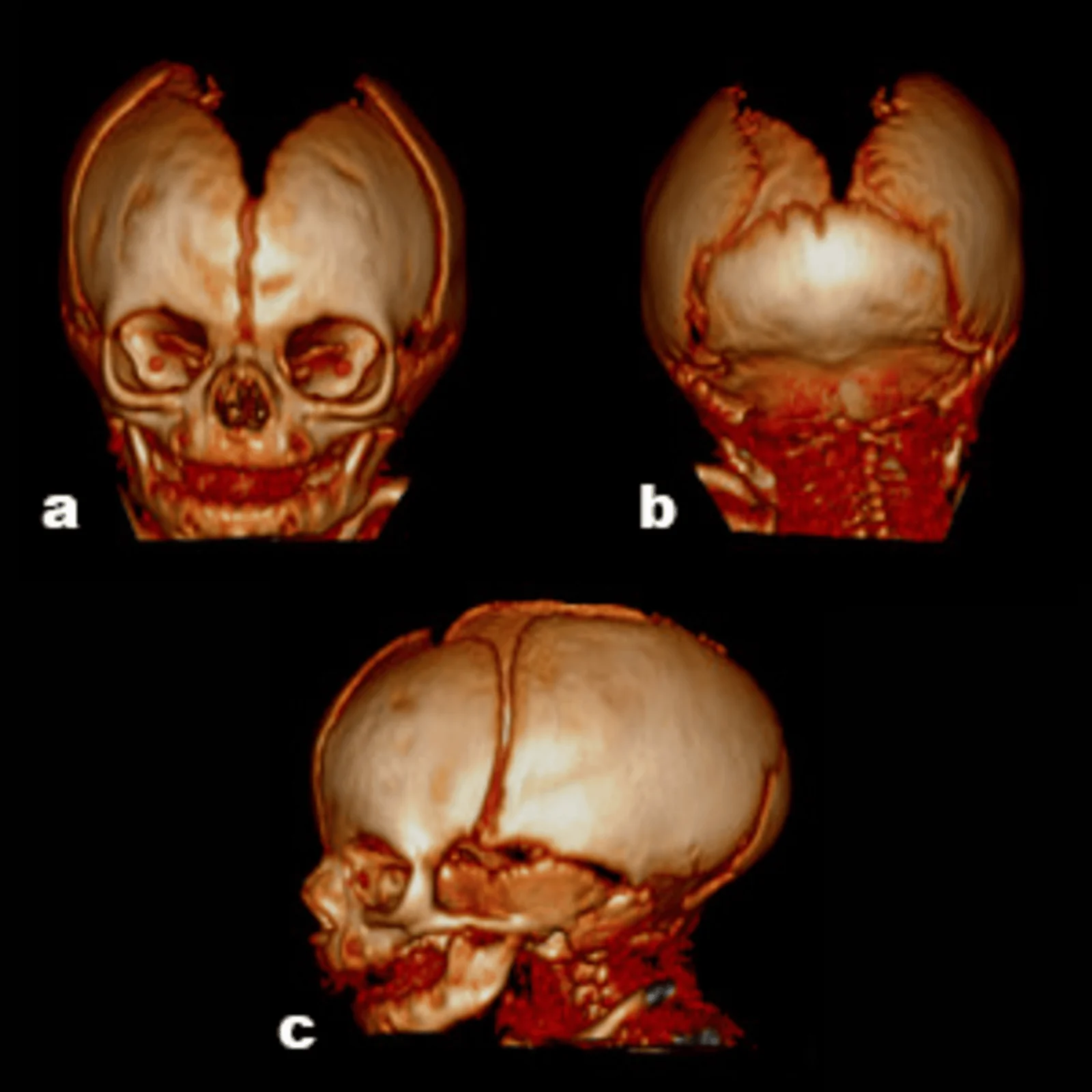
3D reconstruction of cranial CT scan Anterior (a), posterior (b), and profile (c) views, showing the absence of bone tissue at the scalp.

**Figure 3:**
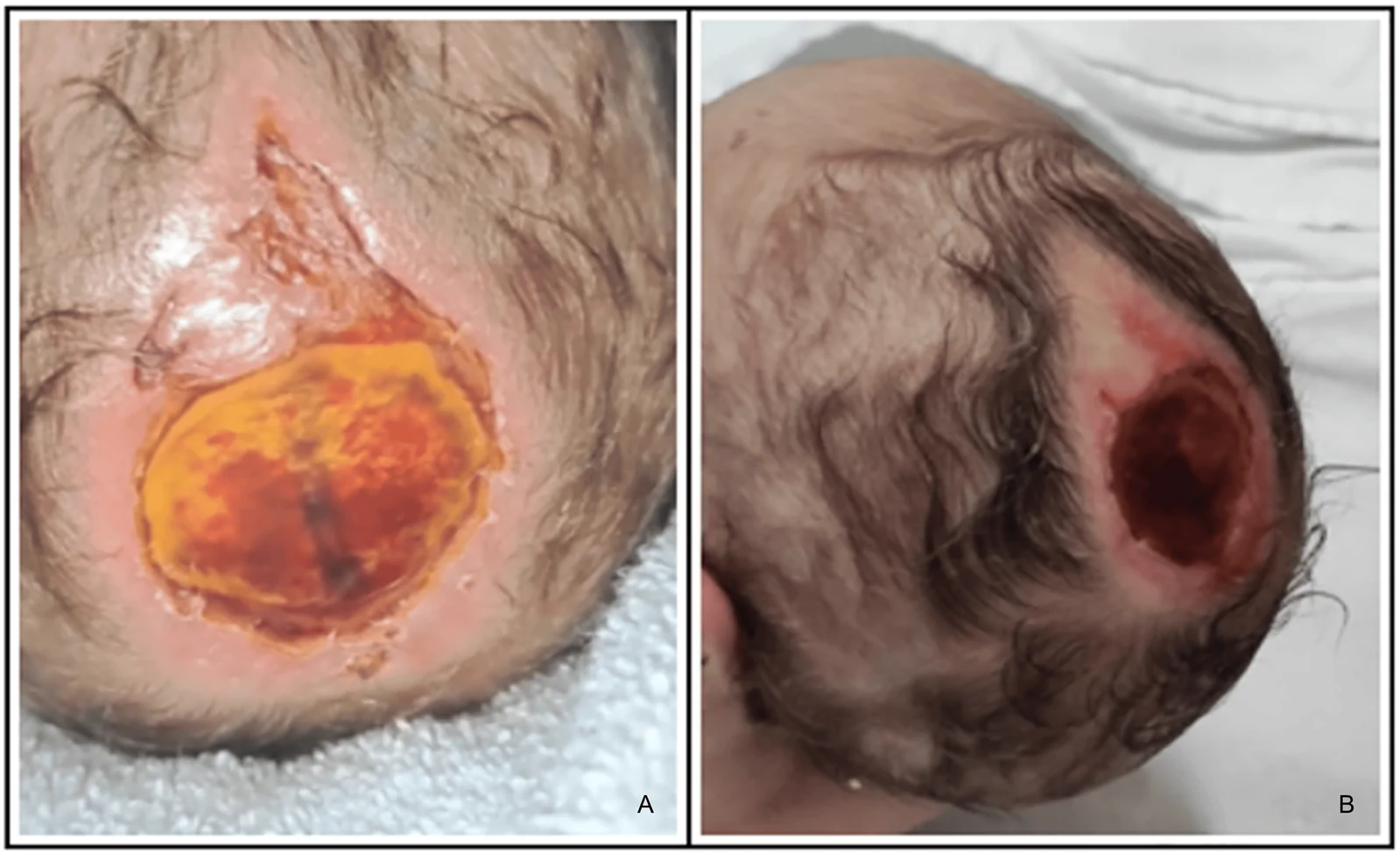
Secondary intention healing of the wound A: fifth day of life; B: ninth day of life

The treatment of the lesion followed the service's Wound Care Committee protocol: the wound was daily cleansed with 0.9% saline solution without friction or drying of the open area, then dressed with 0.9% saline-moistened rayon coated in hydrogel using a sterile technique. The site was occluded with gauze and a bandage secured in a "helmet" fashion to avoid adhesive contact with hair, incorporating local trichotomy if necessary. The case was also discussed with other specialties, such as dermatology, neurosurgery, and plastic surgery teams, who all agreed on a conservative approach and suggested a close clinical follow-up.

## Discussion

ACC is a rare disease that affects the scalp in 96% of cases, characterized by the absence of skin, subcutaneous tissue, and possibly bone tissue [2]. Frieden in 1986 proposed a classification for ACC in nine subtypes, based on concurrent clinical findings (Table 1). Their groupings were later revised and slightly modified, but the main classification remains valid [4,6].

**Table 1:** Frieden's classification of aplasia cutis congenita (1986) Source: [4,6]

Type/Group	Clinical manifestations
1	Isolated lesion of the scalp
2	Scalp lesion with limb malformations (Adams-Oliver syndrome)
3	Aplasia cutis with epidermal nevi
4	Aplasia cutis with overlying embryologic anomalies
5	Aplasia cutis associated with fetus papyraceus or infarcts of the placenta
6	Aplasia cutis with epidermolysis bullosa
7	Aplasia cutis limited to extremities
8	Aplasia cutis with history of exposure to teratogens
9	Aplasia cutis associated with malformation and genetic syndromes

The patient in question can't be classified as isolated ACC due to concomitant physical malformations. The location of the lesion being the scalp and the absence of teratogenic drugs during pregnancy exclude type 7 and 8 ACC. There was no evidence of trauma during pregnancy or obstetric risk factors, such as placental infarcts, substance abuse, or maternal diseases that would lead to embryologic malformations, that could explain the occurrence of aplasia in an intrauterine-complicated scenario, ruling out groups 4 and 5. The lack of obvious skin lesions that would lead to a diagnosis of epidermolysis bullosa and epidermal nevi also excludes our patient from groups 3 and 6.

The partial syndactyly reported in the patient's right foot could be connected to type 2 ACC, but that group has often been linked to Adams-Oliver syndrome (AOS) [4], an extremely rare genetic disorder characterized by aplasia of the scalp, terminal limb reduction, including cutaneous and bone syndactyly, cutis marmorata telangiectasia congenita, and central nervous system anomalies [8,10]. Our newborn did not present any other major clinical manifestations that would be suggestive of AOS, other than cutaneous syndactyly and the aplasia itself, which excludes group 2. 

Although the presentation overlaps with multiple Frieden groups, the type 9 ACC provides the best classification. The case presented had aplasia at the scalp, associated with syndromic-like features, such as low-set ear lobes, mammary and ocular hypertelorism, and facial asymmetry, that can possibly be associated with genetic disorders. Those clinical findings, and the reported autosomal dominant pattern of the disease, could both be related to syndromes known to cause ACC, particularly the SENS. The SENS is an autosomal dominant disorder, characterized by ACC, underdevelopment of nipples and breasts, and anomalies of external ear lobes. Other abnormalities might be present, such as nail dystrophy, cutaneous syndactyly, and renal malformations [7,11].

The SENS is well-known to be a result of mutations in genes that code for KCTD proteins, particularly KCTD [7,11]. Those mutated proteins are the same that were found to be directly involved in scalp ACC's pathomechanism, through experiments in rodents led by Raymundo et al., later in 2023. Raymundo demonstrated that KCTD1 and KCTD15 proteins are responsible for controlling cranial neural crest cell (NCC) functions, and the deletion of both genes in NCC results in scalp ACC in rodents. The study not only uncovered the molecular basis of aplasia cutis and how it is related to SENS but also established scalp ACC as a neurocristopathy [9]. The strong attachment between scalp ACC and KCTD1/15 mutations, along with its autosomal dominant pattern, reinforces the association of our patient's case with genetic syndromes and presents type 9 as the most plausible classification.

Our patient has clinical manifestations that are compatible with SENS, but he doesn’t present all the classical features of the syndrome (Table 2), which would make genetic testing essential for final diagnosis. The exome sequencing of the child and father was requested, but due to the poor economic conditions of the parents and the lack of financial coverage for genetic tests by the Brazilian public healthcare system, those were not performed. The socioeconomic context of an underdeveloped country hinders more in-depth research and implementation due to the high cost of genetic testing, a factor that makes the clinical approach the most viable option. The newborn has a syndromic-like phenotype and a relevant familiar history, which makes type 9 ACC the most plausible, but the lack of exome sequencing, combined with a non-classic pattern of clinical features, makes this classification still uncertain.

**Table 2:** Scalp-Ear-Nipple syndrome manifestations Source: [12]

System/Feature	Clinical manifestations	Frequency	Presented by our patient
Scalp	Aplasia cutis congenita (patchy abnormal areas, firm raised hairless nodules resembling wounds/ulcers at birth, heal with scarring in childhood)	Core feature	Yes
Ears	Small, cup-shaped, folded over, or mildly misshapen external ears; hearing typically normal	Core feature	Yes
Nipples/Breasts	Underdeveloped nipples (hypothelia) or absent nipples (athelia); underlying breast tissue may be absent (amastia)	Core feature	No
Nails	Malformed and brittle fingernails and toenails (nail dystrophy)	Common	No
Digits	Cutaneous syndactyly (fusion of skin between fingers and toes)	Variable	Yes
Dental	Widely-spaced teeth; missing teeth	Variable	Does not apply
Facial	Narrowed palpebral fissures, telecanthus, flat nasal bridge, anteverted nares	Variable	Yes
Renal	Kidney hypoplasia (underdevelopment of one or both kidneys); renal insufficiency	Potentially frequent, requires investigation	No
Ocular	Cataracts	Potentially frequent, requires investigation	To be investigated
Genetics	Autosomal dominant; KCTD1 gene mutations	All cases	To be investigated

Investigation of other malformations with ultrasound tests after birth was performed, regarding the prevalence of 33.8% of associated congenital anomalies found in a European population-based study. The data demonstrated that major limb anomalies (16%) and severe congenital heart defects (15.4%) were the most prevalent in patients with aplasia cutis, followed by nervous system anomalies (7.2%), eye (5.2%), genital (5.2%), urinary (5.0%), and digestive system (4.6%) malformations [2]. The newborn presented an atrial septal defect with no clinical repercussion, and the abdominal and urinary ultrasound did not find any evidence of associated congenital abnormalities. The epidemiological data also showed that 10% of ACC cases are related to chromosomal anomalies, Patau syndrome being the main representative of the group, with approximately 20% of individuals with trisomy 13 manifesting ACC [2,13]. Our patient did not manifest most of the stereotypical features linked to Patau, such as cleft lip and palate, microphthalmia, anophthalmia, microcephaly, polydactyly, abdominal wall defects, or kidney abnormalities. Furthermore, the karyotype revealed a normal 46,XY profile, which rules out this diagnosis [13,14].

Guidelines for management of ACC are somewhat scarce and conflicting in the literature. It is divided into two approaches, the conservative and the surgical, which include the use of grafts or flaps. Several criteria are used to choose which route to follow, but there is no consensus. Early surgical treatment can reduce certain risks, primarily those associated with infection, such as meningitis [15]. However, studies did not show advantages that would justify the surgical risks on a newborn, such as anesthesia-related complications, infections due to manipulation, hemorrhage, and partial or complete loss of the graft, for non-complicated cases, reserving that line of treatment for patients that are unresponsive to conservative care or that present major meningeal defects [3,16].

According to Humphrey et al., conservative management was prioritized in most studies, including cases with larger lesions, such as our case [17]. Secondary intention healing of the wound until complete epithelialization is shown as the most adequate, as long as there is no major involvement of exposed veins and/or direct damage to the meningeal membrane [3]. Bone defects are also shown to heal without surgical intervention after months of clinical management, not being a decisive factor for surgery, due to the dura mater's capacity to stimulate bone growth [15,17]. It is important to consider the risk of sagittal sinus hemorrhage, cerebrospinal fluid leakage, loss of bodily fluid that would lead to dehydration, wound infections, and the increased risk of meningitis, especially when multiple layers of tissue are damaged, highlighting the importance of a multidisciplinary approach [3,15-17].

Our newborn had a 4 cm wound and, although it presented with an exposed dura mater, did not show any signs of complications involving the membrane or major vein exposure that would increase hemorrhage risk. The presented extension of the lesion would make it suitable for surgical intervention according to some references [2,18], but after discussing the case with the neurosurgery, dermatology, and plastic surgery teams of our service, analyzing the risks involved in invasive care on an newborn, and also considering how the father's condition was managed, a conservative approach was chosen, with sterile dressing and minimal manipulation of the wound. After nine days of treatment, until the patient's medical discharge, the conservative management following our service's Wound Care Committee protocol was found to be efficient, considering the absence of local infections and the favorable evolution of the lesion (Figure 4).

**Figure 4:**
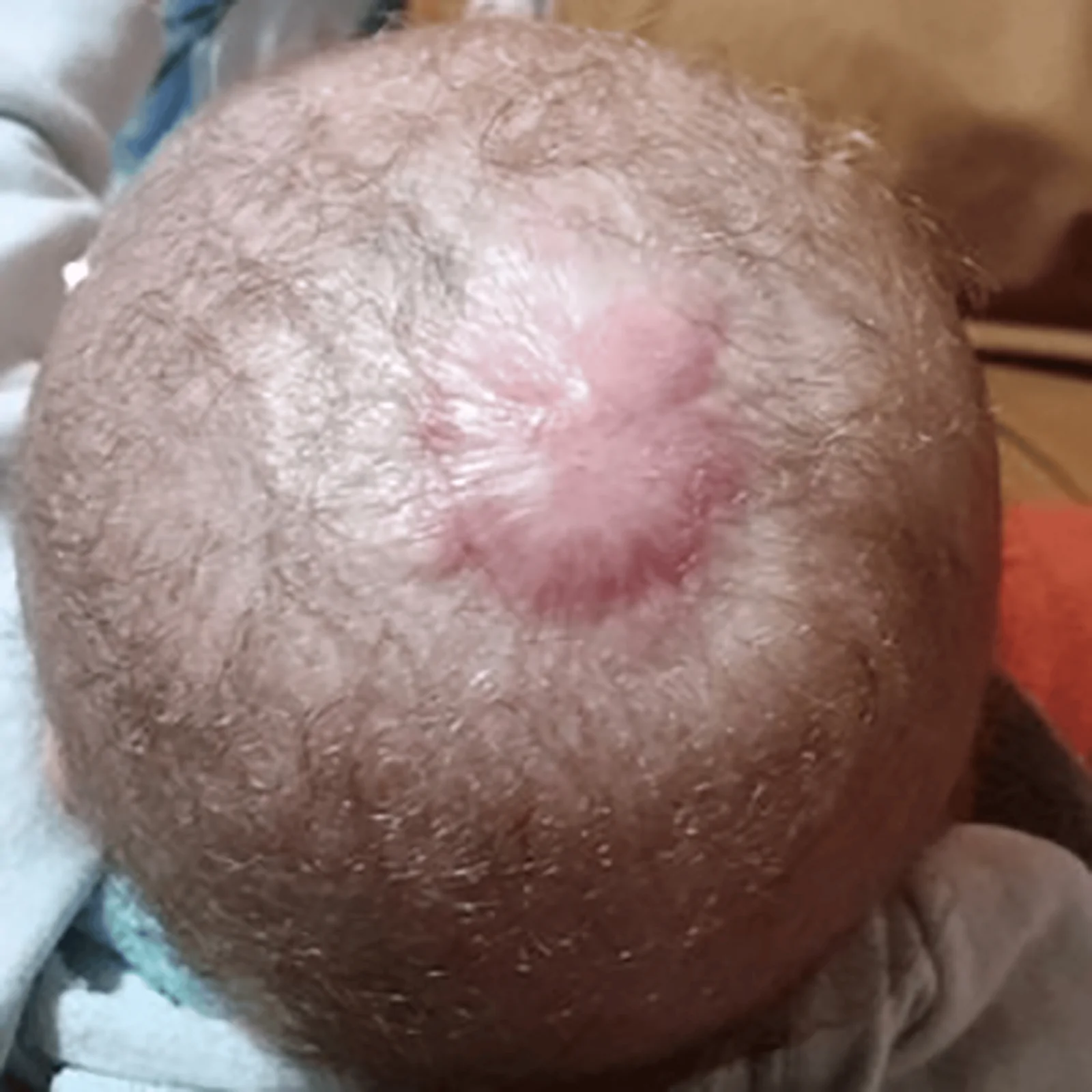
Wound healing progress after three months of conservative treatment

## Conclusions

ACC remains a rare and challenging malformation with a highly variable presentation. In this case, the newborn's syndromic features and strong family history were crucial to identifying a familial pattern, although genetic sequencing would be required for the definitive assessment of the genetic familial risk. Despite the lesion's size and depth, which some guidelines suggest might require surgical intervention, the successful conservative management in this case demonstrates that secondary intention healing is a viable and safe approach. This case emphasizes the necessity of individualized, multidisciplinary care and illustrates how clinical judgment and familial precedents can effectively guide treatment when advanced genetic testing is inaccessible.
